# Engineering Lymphatic Transport of Nanoparticles through Emulsion Polymerization

**DOI:** 10.1021/acsami.5c07771

**Published:** 2025-07-07

**Authors:** Alexander J. Heiler, Tae Hee Yoon, Maya Levitan, Yunus Alapan, Susan N. Thomas

**Affiliations:** † Parker H. Petit Institute for Bioengineering and Bioscience, 1372 Georgia Institute of Technology, Atlanta, Georgia 30332, United States; ‡ School of Chemical and Biomolecular Engineering, Georgia Institute of Technology, Atlanta, Georgia 30332, United States; § George W. Woodruff School of Mechanical Engineering, Georgia Institute of Technology, Atlanta, Georgia 30332, United States; ∥ Wallace H. Coulter Department of Biomedical Engineering, Georgia Institute of Technology and Emory University, Atlanta, Georgia 30332, United States; ⊥ Winship Cancer Institute, Emory University, Atlanta, Georgia 30322, United States

**Keywords:** nanomaterial, transport barriers, synthesis parameters, modeling biomaterial properties, lymphatic targeting, copolymer surfactant

## Abstract

A library of lymphatic-targeting poly­(propylene sulfide) nanoparticles (NPs) synthesized with copolymers of different properties at various concentrations was created. The effects of copolymer type and concentration on NP properties were explored, along with the transport behaviors of the NP formulations using *in vitro* assays that modeled diffusion through the skin interstitium and permeability across and uptake into lymphatic endothelial cells that predicted their *in vivo* lymphatic transport and uptake. By tuning the properties of the copolymer surfactants used during NP synthesis, both the NP characteristics and transport properties could be controlled, enabling the design of lymphatic-targeting drug delivery vehicles and imaging agents with varying behaviors.

## Graphical abstract

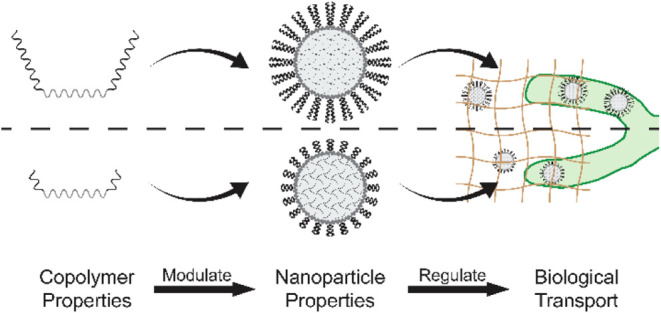

## Introduction

Drug delivery systems (DDSs) are increasingly used to navigate biological barriers to enter lymphatic vessels and drain to the lymph node (LN),^1,2^ which coordinates the commingling of antigens and lymphocytes to mediate both humoral and cell-mediated immune responses.^3,4^ Modulating the interactions between different immune cell types via immunomodulatory therapeutics delivered by LN-targeted DDSs serves to either stimulate the immune response, in diseases such as cancer^5,6^ or infection,^7^ or induce tolerance for autoimmune diseases^8^ or transplanted tissue survival.^9^ Furthermore, imaging tracers, either radiologically or fluorescently labeled, are delivered to the lymphatic system and LN to measure activity, diagnose disease, or inform surgical procedures.^10–12^

However, the transport of both DDSs and tracers, especially nanoparticles (NPs), to the lymphatic system and LN is dependent on the properties of the delivery system.^13–16^ The NP size and surface properties, including poly­(ethylene glycol) (PEG) coatings, influence their transport behavior, such as diffusivity, permeability, drainage from the injection site, accumulation in the LN, and cellular association within the LN.^17–21^ These NP properties are mediated by their synthesis parameters, including the properties of reagents incorporated into the final NP structure, which control the overall NP size, composition, and surface properties, such as the PEG content.^22–25^

Many particle-based DDSs graft PEG onto their surface postsynthesis, including gold,^26^ iron oxide,^27^ quantum dot,^28^ and polymeric systems.^17,18,29,30^ This engraftment allows for great control over the surface properties, including the length and density of the PEG chains, although it requires an additional conjugation reaction. In contrast, some DDSs incorporate PEG as a structural component, such as PEG-containing copolymer hydrogels,^31^ lipid nanoparticles,^32^ and nanoprecipitated copolymers.^9,19,33^ For particle systems synthesized through emulsion polymerization, the surfactant that stabilizes the reaction also becomes incorporated onto the surface of the resulting particle,^34^ and when that surfactant contains PEG, as in the established LN-targeting poly­(propylene sulfide) NPs^22^ utilized in this study, PEG is displayed onto the NP surface without the need for postsynthesis conjugation. These NPs, synthesized using identical reaction conditions while varying only the sizes of polymer blocks within otherwise identical copolymer surfactants, achieve different biological transport properties and serve as a model system for quantifying reagent influences on nanoformulation behavior.

In this work, a suite of compositionally defined PEG-containing copolymers served as emulsion polymerization surfactants to systematically examine how their incorporation into the resulting NPs simultaneously modulated the NP structural properties and their lymphatic-directed transport behavior. The copolymer characteristics that controlled the properties of the bulk NP and their PEG corona also modulated the NP transport trends in *in vitro* transport assays, which were validated through live imaging in an *in vivo* model. These results demonstrate how the properties of NP reagents can be rationally selected to achieve not only desired material characteristics but also transport behaviors ideal for overcoming biological barriers to reach the therapeutically and diagnostically important lymphatic system.

## Experimental Section

### Copolymer Characterization

Pluronic F77, F87, F88, and F98, as well as Tetronic T1107, were provided by BASF. Pluronic F108 and F127 were purchased from Sigma-Aldrich. The critical micellar concentrations (CMC) of the copolymers were measured using pyrene fluorescence as previously described.^35^ Briefly, 50 μL of 1.2 μM of pyrene (Sigma-Aldrich) was incubated for 1 day with 50 μL of the copolymer at different concentrations, with *n* = 3. The ratio of pyrene fluorescence emission at 373 and 383 nm, with a fluorescence excitation at 336 nm, was plotted against the logarithm of the copolymer concentration. The change in slope corresponded to the copolymer CMC.

The hydrophobic–lipophilic balance (HLB) was calculated using equations presented by Guo et al.^36^ Briefly, for Pluronic copolymers, effective polymer chain lengths for the left and right poly­(ethylene glycol) blocks and central poly­(propylene glycol) block, *N*_EO,eff_(left), *N*_EO,eff_(right), and *N*_PO,eff_, respectively, were calculated by using eqs 1 and 2. These effective chain lengths, along with group numbers (GN) presented in Table 1, were used in eq 4 to calculate the HLB. The HLB formula for Tetronic copolymers, eq 5, follows the HLB equation for Pluronic copolymers with the addition of alkyl (eq 3) and tertiary amino groups.

1 $$N_{\mathrm{EO},\mathrm{eff}}=\{\begin{array}{l} 13.45\ln\left(N_{\mathrm{EO}}\right)-0.16N_{\mathrm{EO}}+1.26,1\leq N_{\mathrm{EO}}<50\\ 0.056N_{\mathrm{EO}}+43.08,N_{\mathrm{EO}}\geq 50 \end{array}$$

2 $$N_{\mathrm{P0},\mathrm{eff}}=2.057N_{\mathrm{PO}}+9.06$$

3 $$N_{CH_{2},\mathrm{eff}}=0.965N_{CH_{2}}-0.178$$

4 $${\mathrm{HLB}}_{\mathrm{Pluronic}}=7+{\mathrm{GN}}_{\mathrm{EO}}\times N_{\mathrm{EO},\mathrm{eff}}\left(\mathrm{left}\right)+{\mathrm{GN}}_{\mathrm{EO}}\times N_{\mathrm{EO},\mathrm{eff}}\left(\mathrm{right}\right)+{\mathrm{GN}}_{\mathrm{PO}}\times N_{\mathrm{PO},\mathrm{eff}}+{\mathrm{GN}}_{\mathrm{OH}}+{\mathrm{GN}}_{\mathrm{EtOH}}$$

5 $${\mathrm{HLB}}_{\mathrm{Tetronic}}=7+{\mathrm{GN}}_{\mathrm{EO}}\times N_{\mathrm{EO},\mathrm{eff}}\left(\mathrm{left}\right)+{\mathrm{GN}}_{\mathrm{EO}}\times N_{\mathrm{EO},\mathrm{eff}}\left(\mathrm{right}\right)+{\mathrm{GN}}_{\mathrm{PO}}\times N_{\mathrm{PO},\mathrm{eff}}+{\mathrm{GN}}_{\mathrm{OH}}+{\mathrm{GN}}_{\mathrm{EtOH}}+{\mathrm{GN}}_{CH_{2}}\times N_{CH_{2},\mathrm{eff}}+{\mathrm{GN}}_{N}$$

**Table 1:**
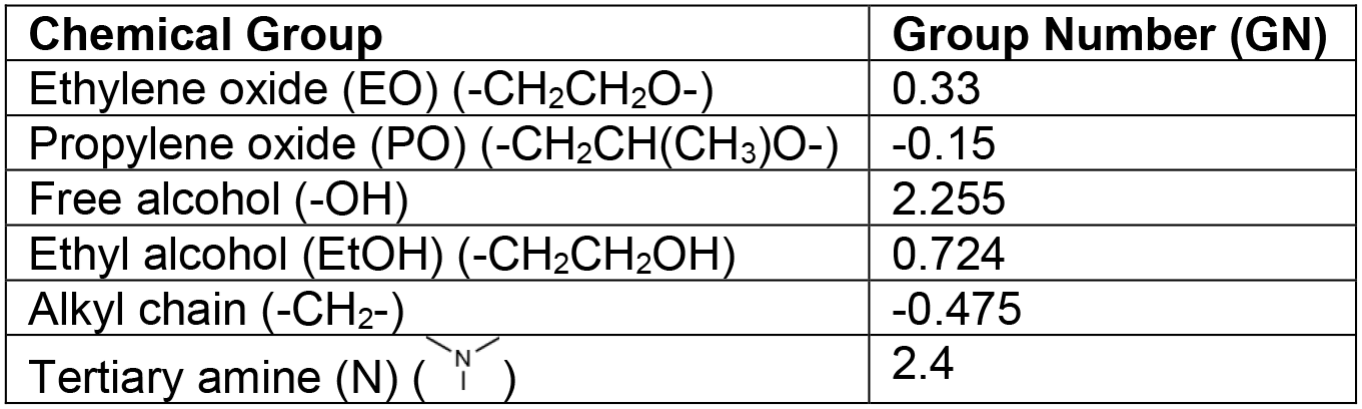
Group Numbers for HLB Calculation

### Poly­(propylene sulfide) Nanoparticle (PPS-NP) Synthesis

PPS-NPs were synthesized as previously described^22,37^ with modifications to the surfactant used and its concentration. Briefly, 1, 5, or 25 wt % (wt%, w/v) of Pluronic or Tetronic copolymer was added to 5 mL of degassed deionized water in a 25 mL two-neck round-bottom flask and stirred at 1500 rpm for 30 min, or until complete dissolution. After degassing again, 200 μL (2.5 mmol) of propylene sulfide (TCI Chemicals) was added under argon for 10 min with stirring. Seven mg (0.02 mmol) of initiator, S,S’-(2,2-bis­((acetylthio)­methyl)­propane-1,3-diyl diethanethioate, was activated with 161 μL of 25 wt % sodium methoxide in methanol (Sigma-Aldrich) for 15 min and added to the round-bottom flask under argon, stirring for an additional 15 min. 32 μL (0.2 mmol) of 1,8-diazabicyclo[5.4.0]­undec-7-ene (Sigma-Aldrich) was added to the reaction mixture under argon and stirred for 24 h. After polymerization, the reaction mixture was exposed to air for 2 h, with stirring, to cross-link NP core thiols. The NP solution was dialyzed against 4 × 5 L of deionized water over 3 days using 100 kDa MWCO cellulose membrane dialysis tubing (Spectrum Lab). Finally, the dialyzed nanoparticle solution was filtered with Acrodisc 0.2 μm filters (VWR).

### NP Characterization

NP hydrodynamic size was measured using dynamic light scattering (DLS) on a Zetasizer Nano ZS instrument (Malvern Panalytical). The NP mass composition was measured using thermogravimetric analysis (TGA, Mettler Toledo TGA2 STAR System) from 200 to 500 °C at a rate of 10 °C/min with a 20 mL/min N_2_ purge. Poly­(propylene sulfide) (PPS) decomposed, on average, at 319 °C, and the copolymer decomposed, on average, at 389 °C. The intermediate mass was defined as the sample mass between the decomposition of PPS and the copolymer. The NP mass composition was determined by eqs 6 and 7:

6 $$\mathrm{PPS}\;\mathrm{composition}=\frac{M_{0}-M_{I}}{M_{I}-M_{f}}\times 100$$

7 $$\mathrm{Copolymer}\;\mathrm{composition}=\frac{M_{I}-M_{f}}{M_{0}-M_{f}}\times 100$$

where *M*_0_ is the initial sample mass, *M*_f_ is the final sample mass, and *M*_I_ is the intermediate mass. The molecular weights of propylene sulfide and the copolymers were then used to determine the NP molecular composition. For the NP characterization, *n* = 1–2.

### NP Stability

NP hydrodynamic size and polydispersity index (PDI) were remeasured by DLS after storage at 4 °C for at least 40 days following synthesis and compared to measurements obtained directly after synthesis, with *n* = 1.

### NP Core Dye Labeling

7.5 μL portion of 10 mg/mL Alexa Fluor 647 C_2_ maleimide (Thermo Fisher) or IRDye 800CW maleimide (LICORbio) in DMSO (VWR) was added to 1 mL of NP solution and mixed for 3 h. Four mg of N-ethylmaleimide (Sigma-Aldrich) was added to the solution to render the remaining core thiols inert. Dye-labeled NP solutions were purified from unreacted dye through dialysis against 4 × 5 L of deionized water over 3 days using 100 kDa MWCO cellulose membrane dialysis tubing and filtering through 0.2 μm Acrodisc filters. The purification of the fluorescent NPs was verified through size exclusion chromatography using Sepharose CL-6B (GE Healthcare) resin.

### Diffusion Assay

Collagen tubes were prepared as previously described.^28,38^ Briefly, 144 μL of 5 mg/mL rat collagen type I (Ibidi) was mixed with 3.7 μL of 1 M NaOH and 19.2 μL of 0.17 M EDTA on ice. The collagen mixture was centrifuged at 200×*g* for 2 min and drawn into 1.0 mm outer diameter capillary tubes (World Precision Instruments) using capillary action. Collagen-loaded tubes were incubated in a 37 °C water bath for 1 h.

NP solutions were loaded into the capillary tubes in contact with the collagen using a 1 mL syringe with a 30-gauge dispensing needle, with *n* = 9–10. Collagen tubes were affixed to a microscope slide, with the tube ends sealed with epoxy, and incubated at 37 °C in a BioTek Cytation 7 for imaging with a Cy5 filter. Diffusion coefficients were calculated by fitting the normalized fluorescence intensity, measured using ImageJ software, to a semi-infinite one-dimensional diffusion model, as shown in eq 8:

8 $$F\left(x,t\right)\propto \mathrm{erfc}\left(\frac{x}{2\sqrt{D_{\mathrm{eff}}t}}\right)$$

### LEC Transwell Assay

Immortalized mouse lymphatic endothelial cells (SV-LEC) were seeded onto Transwell inserts (Sigma-Aldrich) at 3600 cells/mm^2^ and incubated for 1 day at 37 °C to form a monolayer. One mg/mL of Alexa Fluor 647-NP solutions was added to the top of the Transwell, and the concentration of Alexa Fluor 647-NP in the bottom well was measured 4 h later using a Synergy H4 BioTek plate reader, with *n* = 3–6. The effective permeability (*P*_eff_) of the SV-LEC monolayer was calculated according to eq 9:

9 $$P_{\mathrm{eff}}=\frac{V\frac{dC}{dt}}{A\Delta C}$$

where *V* is the volume of the Transwell bottom, $\frac{dC}{dt}$ is the change in NP concentration over time in the Transwell bottom, *A* is the Transwell insert area, and Δ*C* is the difference in NP concentration between the top and bottom of the Transwell. To image the SV-LECs, the Transwell insert was removed from the holder, and the cells were fixed with 4% paraformaldehyde (PFA; Santa Cruz Biotechnology, San Diego, CA, USA) in PBS for 15 min and washed with PBS three times. Nuclei and F-actin were counterstained with 4,6-diamino-2-phenylindole (DAPI; Invitrogen) and rhodamine phalloidin (ActinGreen 488; Invitrogen), respectively. Next, fluorescence images were obtained by using a confocal microscope (Zeiss 900; Carl Zeiss; Oberkochen, Germany). Images were analyzed using ImageJ software, and the Alexa Fluor 647-NP uptake was quantified as the fluorescence intensity of Alexa Fluor 647 divided by the number of cells in the image, normalized by the fluorescence intensity per NP for the respective NP formulation, with *n* = 4–6.

### Lymphatic Drainage of NPs

C57BL/6 mice were purchased from Jackson Laboratories. All animal procedures adhered to guidelines set forth by and were approved by the Institutional Animal Care and Use Committee of the Georgia Institute of Technology. IRDye 800CW-conjugated NPs were intradermally injected into the mouse tail, approximately 1 cm from the tail tip, with *n* = 6 mice per NP formulation. The injection site was imaged for 30 min following the NP injection using an NIR microscope setup as previously described.^39,40^ Briefly, an MVX-ZB10 microscope (Olympus), equipped with a Lambda LS xenon arc lamp (Shutter Instruments), a 769 nm bandpass excitation filter, an 832 nm emission bandpass filter, and an Evolve Delta 512 electron-multiplying charge-coupled device camera (Photometrics), was used to image the injection site by using a 50 ms exposure time at a 10 frames per second capture rate. Analysis started 1000 frames after the start of the video to avoid movement artifacts from the NP injection. The normalized fluorescence (*Y*), measured using ImageJ software, was fit to an exponential decay model following eq 10, with the initial uptake rate (*U*) shown in eq 11:

10 $$Y=\left(Y_{0}-\mathrm{Plateau}\right)e^{-\frac{t}{\tau }}+\mathrm{Plateau}$$

11 $$U=\frac{\left(Y_{0}-\mathrm{Plateau}\right)}{\tau }$$

Additionally, the injection site was reimaged 2 days postinjection, along with the tail lymphatic collecting vessels and excised sciatic lymph nodes.

### Pearson Coefficient

Pearson coefficients were calculated using GraphPad Prism 10. The copolymer concentrations were linearized by taking the logarithm of the copolymer weight %. The data were standardized to a mean of 0 and a standard deviation of 1 prior to analysis. To linearly subtract specific properties from a dataset, a linear model was constructed based on the standardized data for each dependent variable of interest. Then the values of the property to be subtracted were replaced with the mean value of that property, and the dependent variable was recalculated by using the linear model. Therefore, Pearson coefficients could be determined between the recalculated dependent variable and the remaining properties without the influence of the removed property.

### Statistical Analysis

All data are expressed as the mean ± SEM of experimental replicates unless otherwise noted. Statistical analyses were performed in GraphPad Prism 10. Comparisons involving three or more groups were made by one-way ANOVA with Tukey’s multiple comparison test. *p*-values are given as **** *p* < 0.0001, *** *p* < 0.001, ** *p* < 0.01, and * *p* < 0.05. *p*-values are also presented as #### *p* < 0.0001, ### *p* < 0.001, ## *p* < 0.01, and # *p* < 0.05 or $$$$ *p* < 0.0001, $$$ *p* < 0.001, $$ *p* < 0.01, and $ *p* < 0.05.

## Results and Discussion

### Compositionally Distinct Copolymers Form an NP Library Synthesized by Emulsion Polymerization

Poly­(propylene sulfide) NPs^22^ were used as a model system to investigate how the bulk and surface properties of the NP, precisely controlled through the composition of PEG-containing block copolymer surfactants incorporated into the NP corona during emulsion polymerization (Figure 1a), influence their lymphatic-directed transport behavior. Six Pluronic copolymers (F77, F87, F127, F88, F98, and F108) and one Tetronic (T1107), chosen to cover a range of the copolymer properties of interest while still being relatively hydrophilic to promote facile use in aqueous conditions,^41,42^ were used at 1, 5, and 25 wt % to generate a library of NP formulations. While the copolymers were comprised of the same components, i.e., blocks of PEG and poly­(propylene glycol) (PPG) (Figure 1b), they differed in the sizes and relative abundance of the PEG and PPG chains (Figure 1c). The compositional differences resulted in a disparity between the copolymer properties, such as hydrophilicity, measured as the hydrophilic–lipophilic balance (HLB) (Figure 1d), and the room temperature critical micellar concentration (CMC) (Figure 1e). Additionally, the Tetronic was selected to interrogate the architecture of the surfactants as a star copolymer compared to the linear Pluronics. Copolymer concentrations used during synthesis were chosen to correspond to above, below, and around the copolymer CMCs, except for F127, where the copolymer concentrations were comparable to and above its CMC. Twenty-five wt % NP formulations composed of F127, F98, and F108 were unable to be synthesized because the copolymers formed hydrogels after dissolution, as 25 wt % is above their room temperature critical gel concentrations.

**Figure 1:**
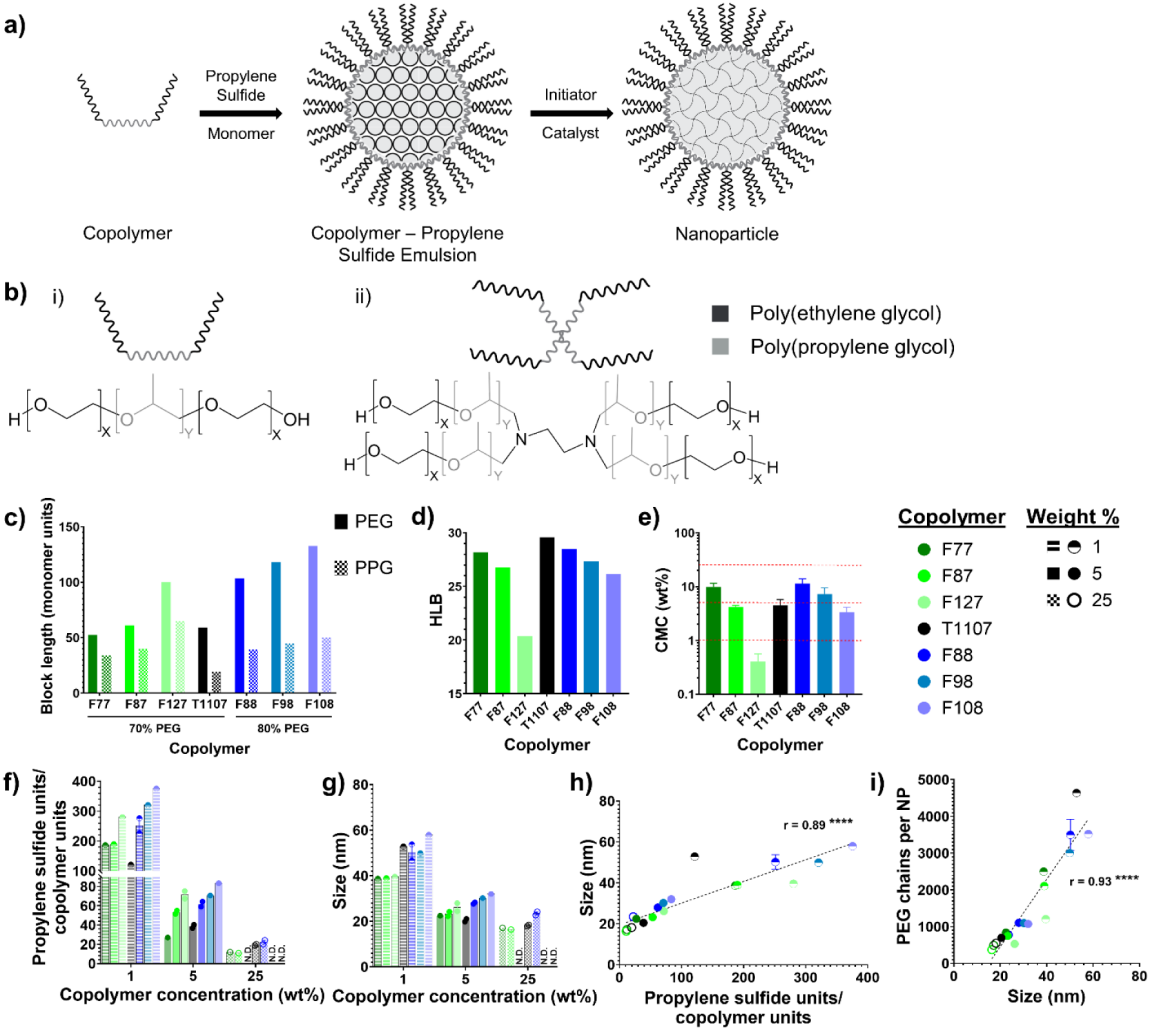
Copolymer surfactant modulates the nanoparticle material properties. (a) Schematic of emulsion polymerization from the copolymer surfactant to NP formation. (b) Schematic of (i) Pluronic and (ii) Tetronic copolymers, with X and Y, respectively, referring to the poly­(ethylene glycol) and poly­(propylene glycol) block lengths in c. (c) Size of the poly­(ethylene glycol) and poly­(propylene glycol) polymer blocks in each copolymer. (d) Calculated hydrophilic–lipophilic balance (HLB) of the copolymers. (e) Critical micellar concentration (CMC) of the copolymers (*n* = 3). Red lines indicate the copolymer concentrations used during NP synthesis. (f) NP composition as the ratio of propylene sulfide monomer units to the copolymer molecules per NP for NPs synthesized with varying copolymers (*n* = 1–2). (g) Hydrodynamic size by volume of NPs measured by dynamic light scattering (*n* = 1–2). (h) Comparison of NP size and composition. (i) Number of PEG chains per NP dependence on NP size. Dotted lines represent linear regression with Pearson correlation coefficient (*r*), and ***** indicates the slope significantly deviates from zero. **** *p* < 0.0001, *** *p* < 0.001, ** *p* < 0.01, and * *p* < 0.05.

After synthesizing a library of NP formulations based on different copolymers and copolymer concentrations, the NP properties were characterized. Thermogravimetric analysis measured the NP constitution based on mass (Figure S1), which was converted to a molecular composition as the ratio of the core propylene sulfide units to coronal copolymer units (Figure 1f), as well as the extent of PEG incorporation (Figure S2b). Notably, the NP composition segregated based on the copolymer concentration used during synthesis, with increasing copolymer concentrations resulting in a smaller ratio of propylene sulfide units to copolymer units, yielding a smaller core relative to the copolymer corona. Furthermore, the NP hydrodynamic size, measured using dynamic light scattering (DLS) (Figure 1g), also partitioned based on the copolymer concentration. As expected, increasing the surfactant concentration resulted in smaller particles. A higher copolymer concentration is able to encapsulate a larger surface area of propylene sulfide, which is achieved by forming an increased number of smaller emulsions that result in smaller NPs with a decreased propylene sulfide-to-copolymer ratio,^22^ corroborating how the size and composition of the NPs were directly proportional to each other (Figure 1h). Notably, the 1 wt % NPs had sizes and compositions much greater than the other NPs, with a much larger range for either property. Because 1 wt % is below most of the copolymer CMCs, micelles were not formed prior to the introduction of the monomer, causing a shift from microemulsion to miniemulsion polymerization.^34,43^ This change in the emulsion polymerization mechanism likely caused not only the significant increase in compositional ratio and size but also the increased variability in these properties. Additionally, the NP surface incorporates PEG through the copolymer surfactants, and the amount of PEG chains per NP increased with the NP size (Figure 1i). While larger NPs had fewer copolymers relative to the propylene sulfide core, they still had a larger total amount of copolymers forming the corona (Figure S2a) and therefore more PEG chains comprising the NP surface (Figure S2b). Overall, changing the copolymer concentration caused large variations in the bulk and surface properties of the NPs.

### Copolymer Properties Differentially Mediate the NP Structural Properties

Nevertheless, the copolymer library yielded NPs with differences in properties beyond the expected variation from different copolymer concentrations. Therefore, differences in copolymer properties were investigated to understand how they drive variations in NP properties. When compared to the PEG chain length within the copolymer used during synthesis, both the compositional ratio and the NP size generally increased with an increase in PEG chain length (Figure 2a,b). However, the size of the PEG block in the copolymers did not affect all NP properties equally, as evidenced by the poor correlation between the PEG chain length and the number of PEG chains per NP (Figure 2c). Even though the PEG chain length is a crucial property for influencing NP properties, it is not responsible for all the differences seen between the NP formulations, warranting further investigation into other copolymer properties that mediate NP material properties.

**Figure 2:**
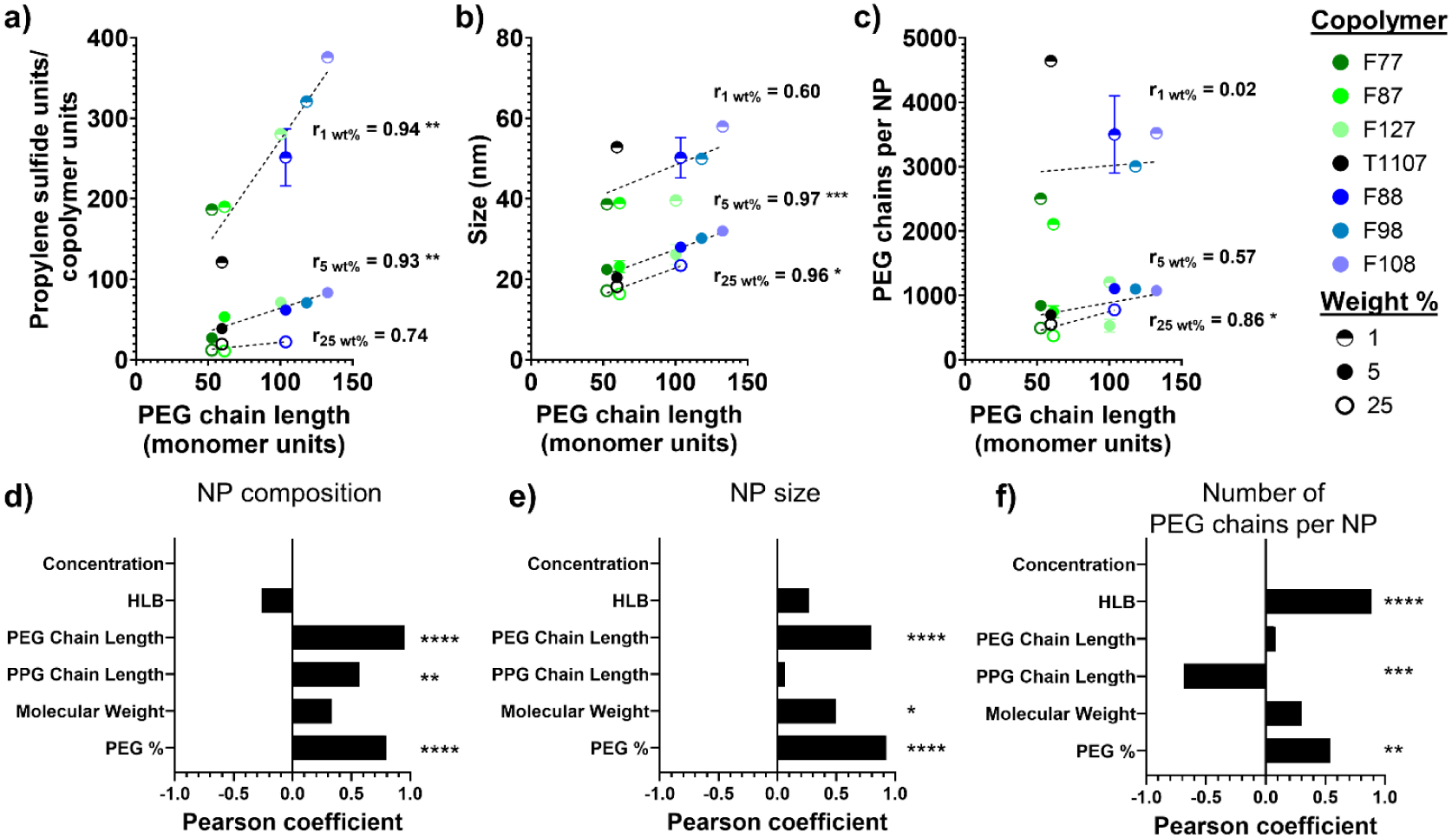
Copolymer properties mediate the nanoparticle properties. (a–c) a) Ratio of propylene sulfide monomer units to the copolymer molecules, (b) NP hydrodynamic size, and (c) PEG chains per NP compared to the PEG chain length in the copolymer corona. Dotted lines represent linear regression for the respective wt % group with Pearson correlation coefficient (*r*), and * indicates the slope significantly deviates from zero. (d–f) Pearson coefficient of the NP properties: (d) composition (propylene sulfide units per NP/copolymer units per NP), (e) size, and (f) PEG chains per NP versus the copolymer properties, with the copolymer concentration linearly removed from the NP properties. * represents significant correlation. **** *p* < 0.0001, *** *p* < 0.001, ** *p* < 0.01, and * *p* < 0.05.

In order to investigate the influence of copolymer properties on the NP characteristics , correlations between the copolymer properties and each NP property of interest were evaluated. As expected, copolymer concentration was the most correlated variable for NP composition, size, and the number of surface PEG chains (Figure S3). Copolymer concentration was also negatively correlated, indicating that an increase in copolymer concentration decreases the compositional ratio, size, and number of PEG chains per NP, in agreement with experimental trends.

Because the copolymer concentration was much more correlated than the other copolymer properties, a linear model for each NP property was constructed and used to remove the dominating influence of the copolymer concentration to better analyze the copolymer properties in relation to the NP properties. For both the NP composition and size, the PEG chain length and percentage of PEG in the copolymer were the two most correlated variables and positively correlated (Figure 2d,e). When taken together, they describe the amount of PEG in the surfactant. More hydrophilic PEG likely allows the emulsion to encapsulate more hydrophobic propylene sulfide during synthesis, leading to an increased propylene sulfide-to-copolymer ratio, a relatively larger core structure, and a larger overall NP size. Furthermore, because the PEG chains reside on the NP surface, more PEG in the copolymers likely increases the size of the copolymer corona and, therefore, the overall NP. In agreement with the previous trends for the number of PEG chains per NP, the PEG chain length was less correlated, with the HLB and PPG chain length as the two most correlated variables (Figure 2f). The PPG length was negatively correlated, demonstrating that shorter PPG chains, which connect and anchor the PEG blocks, translate to tighter packing of the PEG chains and thus more PEG chains on the NP surface. Furthermore, the HLB, which was positively correlated, can be seen as the ratio of the hydrophilic PEG units to the hydrophobic PPG units. Therefore, a larger HLB indicates that more PEG relative to PPG also leads to tighter packing of the PEG chains. Overall, the properties of the copolymer surfactant can mediate the surface coverage of PEG on the NPs in addition to their composition and size.

The stability of the NPs formed using different synthesis parameters was also evaluated. NPs were stored at 4 °C for at least 40 days and remeasured using DLS to determine the extent of changes to their hydrodynamic size and polydispersity index (PDI) (Figure S4). In general, the 1 wt % NPs were stable upon storage, with minimal changes to either the size or PDI, whereas the NPs with copolymers consisting of 70% PEG were less stable at higher copolymer concentrations. The relatively larger cross-linked PPS core of the 1 wt % formulations likely stabilizes the NP, while the higher percentage of PEG in the copolymer likely reduces aggregation.

### NP Diffusivity through Collagen Is Mediated by the Copolymer Properties Important for Controlling the NP Properties

Having characterized the NP properties in the context of the copolymer properties, the transport of the NPs was then evaluated using *in vitro* assays. To model NP delivery through the skin interstitium, a collagen diffusion assay based on the setup described in Wong et al.^28^ and employed in multiple studies^20,38,44^ was utilized (Figure 3a–c). A subset of NPs was chosen to span a representative range of the copolymer and NP properties (Figure 3d), and the non-cross-linked thiols of the NP core were labeled with an Alexa Fluor 647 fluorophore to image the NP movement through the collagen using fluorescent imaging (Figures S5 and 3a–c). Finally, the fluorescence intensity was fit to a 1D diffusion model to determine the effective diffusion coefficient (*D*_eff_) (Figure 3e).

**Figure 3:**
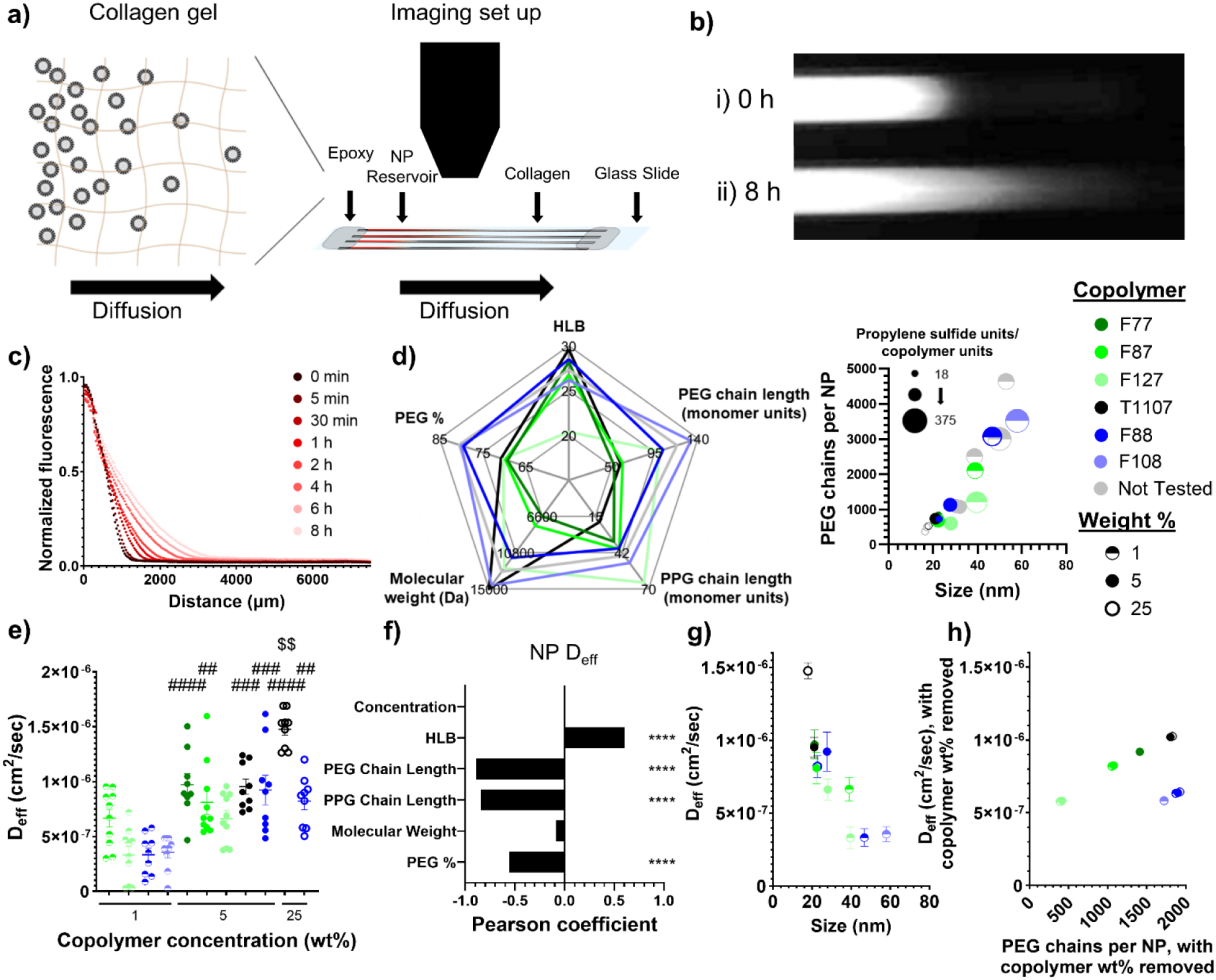
NP structure and copolymer property effects on diffusion through a collagen gel. (a) Schematic of NP diffusion through collagen hydrogel, imaged using a fluorescent microscope. (b) Representative images of NPs diffusing through a collagen gel after (i) initial setup and (ii) 8 h. (c) Representative graph of the normalized fluorescence of NPs beyond the NP reservoir for 5 wt % F87NP. (d) Copolymer and NP properties of nanoparticle formulations used in the diffusion assay. (e) Effective diffusion coefficients determined from fitting the normalized NP fluorescence to a one-dimensional diffusion model (*n* = 9–10). $$ indicates significant difference (*p* < 0.01) between the designated NP formulation and all other NP formulations, whereas ##, ###, and #### (*p* < 0.01, *p* < 0.001, and *p* < 0.0001, respectively) indicate significant difference between the designated NP formulation and the 1 wt % F127NP, 1 wt % F88NP, and 1 wt % F108NP formulations by one-way ANOVA with Tukey’s multiple comparison test. (f) Pearson coefficient of the NP diffusivity versus the copolymer properties, with the copolymer concentration linearly removed, and * representing significant correlation. **** *p* < 0.0001, *** *p* < 0.001, ** *p* < 0.01, and * *p* < 0.05. (g) Comparison of effective diffusion coefficients and the NP size. (h) Comparison of effective diffusion coefficients and the number of PEG chains per NP, with the copolymer concentration linearly removed from both.

Investigating the influence of the copolymer properties on NP diffusion, the copolymer concentration was most correlated to *D*_eff_ (Figure S6), driving the major differences in NP diffusivity, similar to the major copolymer concentration-dependent disparities within the NP properties. After using a linear model to remove the concentration dependence, the copolymer properties most correlated with NP diffusivity were the HLB, PEG chain length, PPG chain length, and percentage of PEG in the copolymer (Figure 3f), which were also highly correlated with the NP properties. When compared to the NP properties, the NP diffusivity was largely mediated by the NP size (Figure 3g), following established trends of decreased diffusivity through hydrogels for larger samples, including proteins^38^ and small molecules.^45^ Both the PEG chain length and percentage of PEG in the copolymer were shown to be negatively correlated with the NP size, suggesting that less PEG in the copolymers, which results in smaller NPs, increases diffusivity. Additionally, lower-molecular-weight PEG coatings have been shown to improve NP diffusion through mucus and Matrigel, as higher-molecular-weight PEG becomes entangled with the mucus and gel meshes.^29,30^ Therefore, shorter PEG chains likely form fewer entanglements with the collagen fibrils as the NPs move through the collagen pores, further improving diffusivity. A positive correlation with HLB and a negative correlation with PPG chain length, which were correlated with an increasing number of PEG chains per NP, suggest that a higher surface coverage of PEG increases diffusivity (Figure 3h). More PEG chains forming the NP corona likely better shield the NP and diminish interactions between the NP and the collagen matrix, increasing diffusivity, in agreement with trends established by Ramirez et al.^17^ Overall, the HLB, PEG length, PPG length, and PEG percentage, all important copolymer parameters for modulating the NP properties, also mediated the diffusivity of the NPs through the collagen. Therefore, controlling the composition of the copolymer surfactant used during NP synthesis enables control over not only the NP properties but also their transport behavior.

### Lymphatic Endothelial Cell Permeability and Uptake Inversely Depend on PEG Content

To further explore the influence of copolymer properties on NP delivery, the transport of NPs across a lymphatic endothelial cell (LEC) monolayer was evaluated as a model of uptake by the initial lymphatics (Figure 4a). After incubating LECs with the same subset of NPs used to evaluate the effects of copolymer properties on NP diffusivity (Figure 3d), the effective permeability (*P*_eff_) was determined from the concentration of NPs transported across the LEC monolayer, while endocytosis into the LECs was determined from confocal microscopy images of the LECs (Figure 4b). The 25 wt % NPs exhibited the highest permeability across the LECs (Figure 4c), with decreasing permeability observed for the 5 and 1 wt % NPs, demonstrating that a higher copolymer concentration, which results in smaller NPs, increased permeability (Figure S7a,c). After the influence of copolymer concentration was removed, the PEG chain length, copolymer molecular weight, and percentage of PEG in the copolymer were identified as the most correlated copolymer properties to the NP permeability (Figure 4d). Taken together, these properties describe the amount of PEG in the copolymer, indicating that increased amounts of PEG increased the transport of NPs across LECs. Because PEG is often used to inhibit interactions between biomaterials and cells,^27,46^ increased PEG in the copolymer—and thus the NP—reduced interactions between the NPs and the LECs, allowing the NPs to move across the monolayer less impeded. Notably, for the NPs synthesized using 5 wt % copolymer, their diffusivity and permeability were inversely correlated (Figure 4e), demonstrating that the overall amount of PEG in the copolymer has counteracting influences on NP transport.

**Figure 4:**
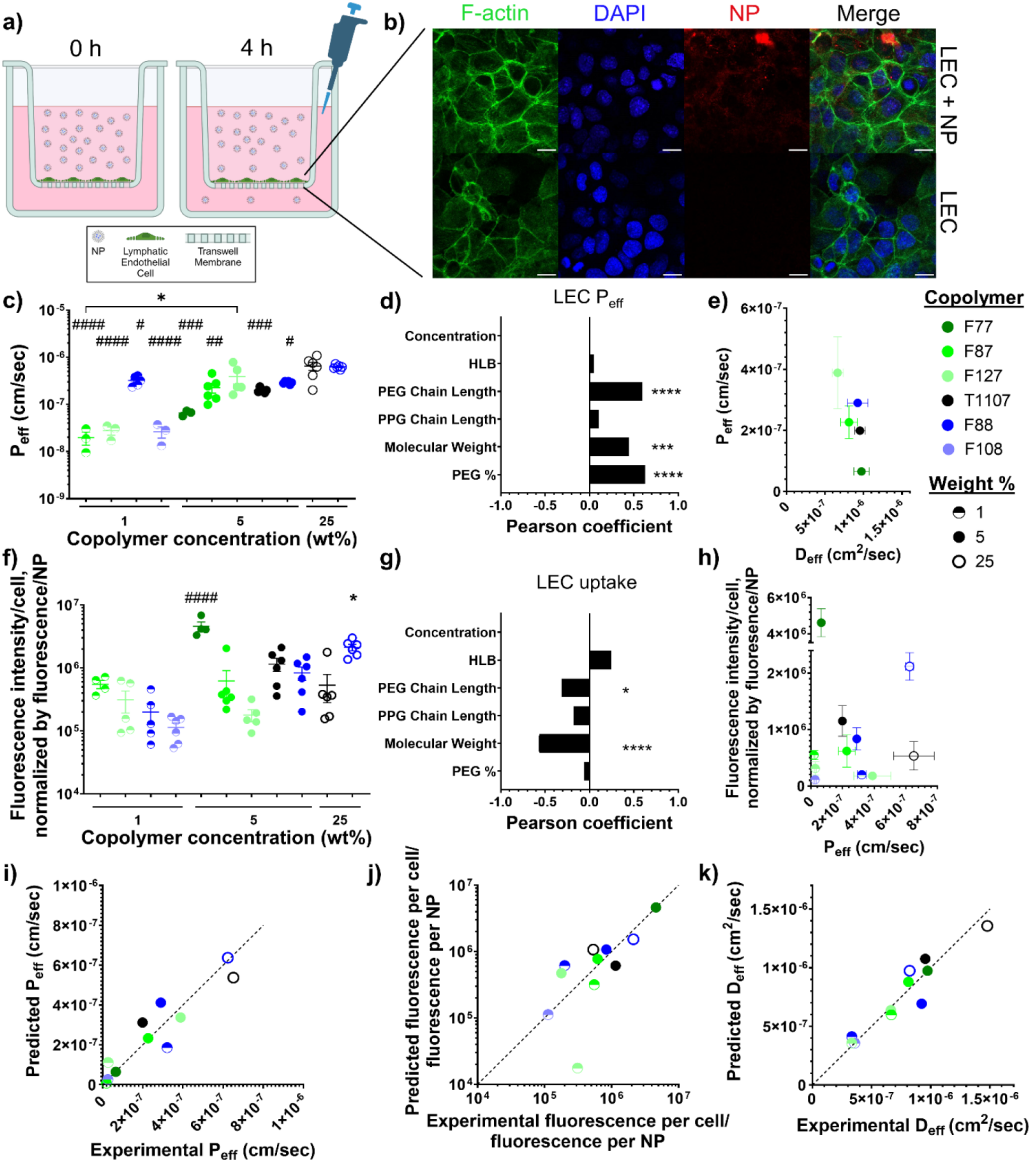
Transport across LEC monolayer dependence on NP and copolymer properties. (a) Diagram of Transwell setup, with an LEC monolayer seeded on the Transwell membrane. (b) Representative confocal microscopy images of LECs incubated on a Transwell membrane, with or without NPs, for 4 h at 37 °C. Scale bars indicate 15 μm. (c) Permeability of NP formulations across the LEC monolayer after 4 h incubation at 37 °C (*n* = 3–6). * indicates significant difference between the indicated NP formulations, while #, ##, ###, and #### indicate significant difference between the indicated NP formulation and both 25 wt % F88NP and 25 wt % T1107NP by one-way ANOVA with Tukey’s multiple comparison test. (d) Pearson coefficient of the LEC permeability versus the copolymer properties, with the copolymer concentration linearly removed. * represents significant correlation. (e) LEC permeability versus diffusivity for 5 wt % NPs. (f) LEC uptake of NPs after incubation for 4 h at 37 °C as the NP fluorescence intensity per cell, normalized by fluorescence intensity per NP (*n* = 4–6). * indicates significant difference between the indicated NP and all other NP formulations except 5 wt % T1107NP, while #### indicates significant difference between the indicated NP and all other NP formulations by one-way ANOVA with Tukey’s multiple comparison test. (g) Pearson coefficient of the LEC uptake versus the copolymer properties, with the copolymer concentration linearly removed. * represents significant correlation. (h) LEC uptake dependence on the LEC permeability. (i–k) Predicted values, using linear models based on the experimental data, versus the experimental values for (i) LEC permeability, (j) LEC uptake, and (k) NP diffusivity. Dotted line signifies complete agreement between predicted and experimental values. **** *p* < 0.0001, *** *p* < 0.001, ** *p* < 0.01, and * *p* < 0.05, and #### *p* < 0.0001, ### *p* < 0.001, ## *p* < 0.01, and # *p* < 0.05.

Next, the retention of NPs through LEC endocytosis was measured (Figure 4b,f) as a competing influence slowing the NPs’ ability to cross the LEC monolayer. Smaller NPs tended to have decreased LEC uptake, which agrees with the positive correlation to the copolymer concentration (Figure S7b,d). After the copolymer concentration dependence was removed, the PEG chain length and copolymer molecular weight were the most correlated copolymer properties to LEC endocytosis (Figure 4g). The negative correlation indicates that smaller copolymer chains with less PEG increase LEC uptake, corroborating the notion of using high-molecular-weight PEG chains to decrease cellular endocytosis.^47^ Interestingly, 1 wt % NPs exhibited both low permeability and uptake, indicating that low copolymer concentrations reduce transport in general (Figure 4h). For the 5 and 25 wt % NPs, the uptake and permeability had an inverse relationship, with highly permeable NPs showing decreased uptake, such as the 25 wt % T1107NP and 5 wt % F127NP, while less permeable NPs exhibited increased uptake, such as the 5 wt % F77NP.

Beyond showcasing trends in NP transport, the constructed linear models for NP permeability, uptake, and diffusivity were evaluated for estimating the NP transport behavior based on their synthesis parameters, including the properties and concentration of the copolymer used during emulsion polymerization. There was high agreement between the values predicted by the models and the experimental values (Figure 4i–k), demonstrating that the composition of the copolymers directly modulates the NPs’ responses in assays for lymphatic delivery. Therefore, the copolymer properties can be rationally selected before NP synthesis to achieve the desired transport behavior.

### *In Vitro* Transport Trends Translate to *In Vivo* Lymphatic Transport

To test the accuracy of the model predictions and the translatability of the *in vitro* trends to *in vivo* models, near-infrared (NIR) fluorophore-labeled 25 wt % T1107NP and 5 wt % F127NP, which exhibited high permeability and low endocytosis, along with the 5 wt % F77NP, which had low permeability, high endocytosis, and a comparable diffusivity to 5 wt % F127NP, were injected into the tail tips of mice to measure their *in vivo* performance (Figure 5a–c). The NIR-NPs showed an exponential decrease in normalized fluorescence at the injection site as the NPs drained into the lymphatic system, through the collecting vessels, and toward the sciatic LNs (Figure 5b,c). In comparison to the 5 wt % F77NP, the 5 wt % F127NP and 25% T1107NP demonstrated faster overall uptake into the lymphatic system (Figure 5d–f), which was predicated on their higher *in vitro* permeability across the LEC monolayer (Figure 5g–i). Drainage from the injection site of lymphatic-directed DDSs has been shown to increase with the increasing molecular weight of their PEG coatings,^19^ corroborating the increase in lymphatic uptake of the 5 wt % F127NP and 25 wt % T1107NP. After 2 days, all the NIR-NPs drained from the injection site to similar extents (Figure 5j), likely due to a combination of passive drainage and active cellular processing from peripheral antigen-presenting cells^4,48^ that is not dependent on the NP properties over longer time scales.

**Figure 5:**
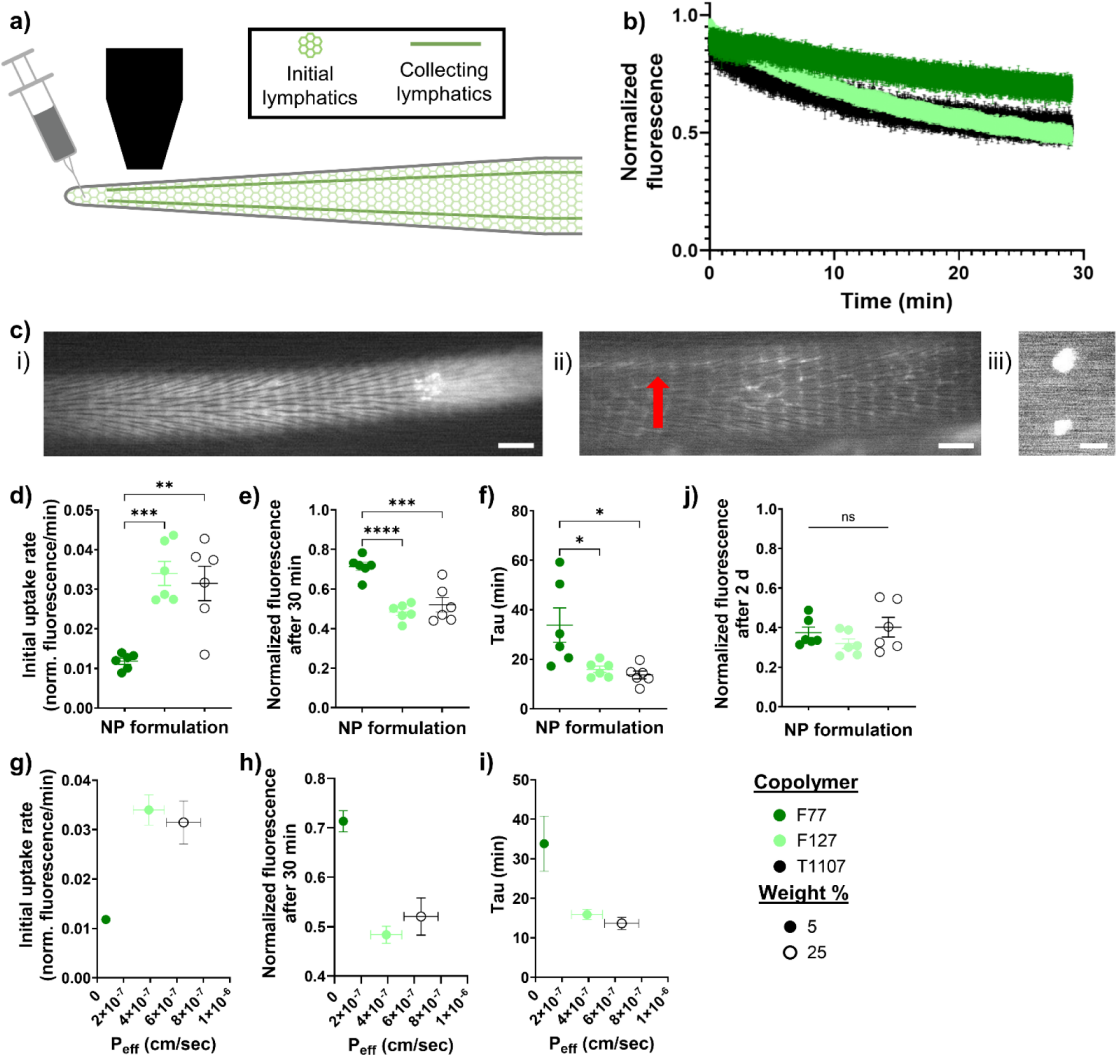
Differential NP drainage into lymphatic vessels. (a) Schematic of NIR-labeled NP uptake into mouse tail lymphatics after subcutaneous injection. (b) Normalized fluorescence of NIR-NPs at injection site immediately following subcutaneous injection. (c) Representative NIR images of (i) mouse tail, (ii) collecting vessel, and (iii) sciatic LNs after NIR-NP injection. White represents NP fluorescence at 769 nm excitation and 832 nm emission, while the red arrow designates the collecting vessel. Scale bars indicate 1 mm. (d–f) Initial uptake rate, (e) normalized fluorescence after 30 min, and (f) tau of NIR-NP uptake into mouse tail lymphatics after subcutaneous injection. *, **, ***, and **** indicate significant difference between the indicated NP formulations by one-way ANOVA with Tukey’s multiple comparison test. (g–i) Comparison of NIR-NP: (g) initial uptake rate, (h) normalized fluorescence after 30 min, and (i) tau with NP permeability *in vitro*. (j) Normalized fluorescence of NIR-NP injection at injection site after 2 d. *n* = 6 mice per group, with **** *p* < 0.0001, *** *p* < 0.001, ** *p* < 0.01, and * *p* < 0.05.

## Conclusion

This study constructed quantitative models to evaluate the influence of emulsion-mediated PEG characteristics on both the NP properties and transport behavior related to lymphatic system-directed delivery. The NP transport behavior was demonstrated to depend on the amount of PEG and the distance between PEG chains within the copolymer surfactants, enabling the design of NPs with desired properties for specific *in vivo* applications at the synthesis level without the need for postsynthesis modifications. Nanoformulation tracers entered the mouse tail lymphatic system following trends established by *in vitro* assays, validating the modulation of NP transport through control over the reagent properties. Due to the high translatability of NP transport behavior from *in vitro* to *in vivo* based on the assembled models, this method of model construction may be applied to other NP systems to quantify reagent influences on nanoformulation behavior.

## Supplementary Material



## Data Availability

Data pertaining to nanoparticle characterization and transport assays are available upon request from the authors.

## Notes

Thermogravimetric analysis for nanoparticle composition, Pearson coefficients prior to removing copolymer concentration via linear models, nanoparticle stability, fluorescent nanoparticle characterization (PDF)
