# Supplementary material for: Engineering Lymphatic Transport of Nanoparticles through Emulsion Polymerization
Source: ACS Appl Mater Interfaces. 2025 Jul 7;17(28):40173–83. doi: 10.1021/acsami.5c07771 (PMC12278220; doi:10.1021/acsami.5c07771)
Supplement: Supplementary file 1 [file am5c07771_si_001.pdf]

Supporting Information

Engineering lymphatic transport of nanoparticles by their emulsion polymerization

Alexander J. Heiler[1,2], Tae Hee Yoon[1,3], Maya Levitan[1,4], Yunus Alapan[1,3], and Susan N. Thomas[1,3,4,5*]

[1]Parker H. Petit Institute for Bioengineering and Bioscience, Georgia Institute of Technology, Atlanta, GA 30332, United States of America

[2]School of Chemical and Biomolecular Engineering, Georgia Institute of Technology, Atlanta, GA 30332, United States of America

[3]George W. Woodruff School of Mechanical Engineering, Georgia Institute of Technology, Atlanta, GA 30332, United States of America

[4]Wallace H. Coulter Department of Biomedical Engineering, Georgia Institute of Technology and Emory University, Atlanta, GA 30332, United States of America

[5]Winship Cancer Institute, Emory University, Atlanta, GA 30322, United States of America

*Corresponding Author:

Susan N. Thomas, PhD.
Georgia Institute of Technology
IBB 2310
315 Ferst Drive NW
Atlanta, GA 30332
United States of America
404-385-1126
susan.thomas@gatech.edu

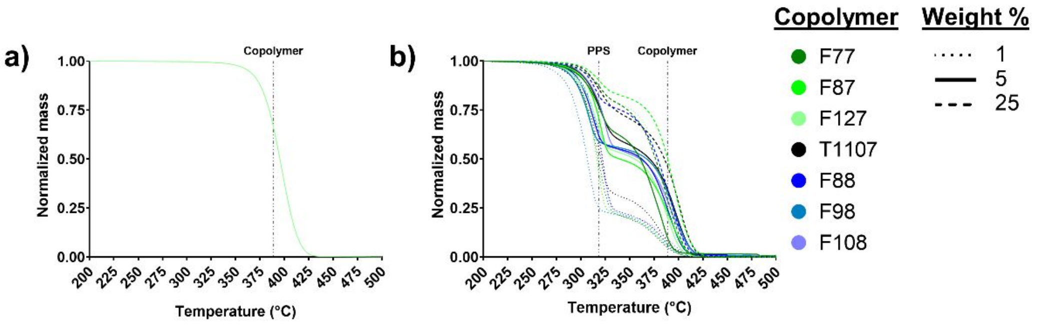

Figure S1. Thermogravimetric analysis of the NP formulations. a) Normalized decrease in mass with increasing temperature for Pluronic F127, measured by thermogravimetric analysis. b) Normalized decrease in mass with increasing temperature for each NP formulation, measured by thermogravimetric analysis. Vertical dotted lines represent the decomposition temperature for poly(propylene sulfide) (PPS) and the copolymers.

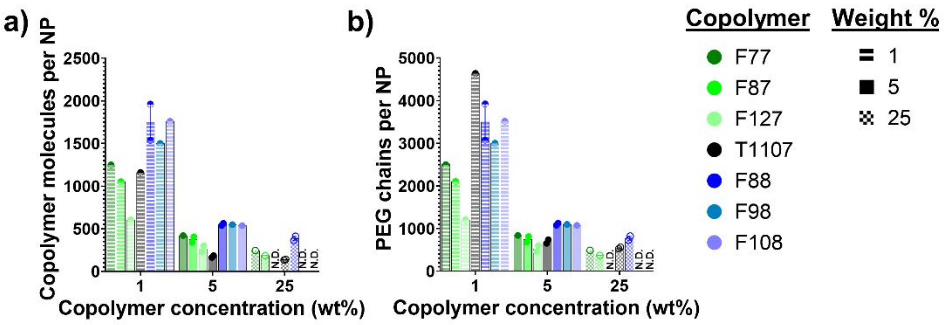

Figure S2. Nanoparticle corona composition. a) Number of copolymers forming the corona of each NP formulation. b) Number of PEG chains forming the corona of each NP formulation (n=1-2).

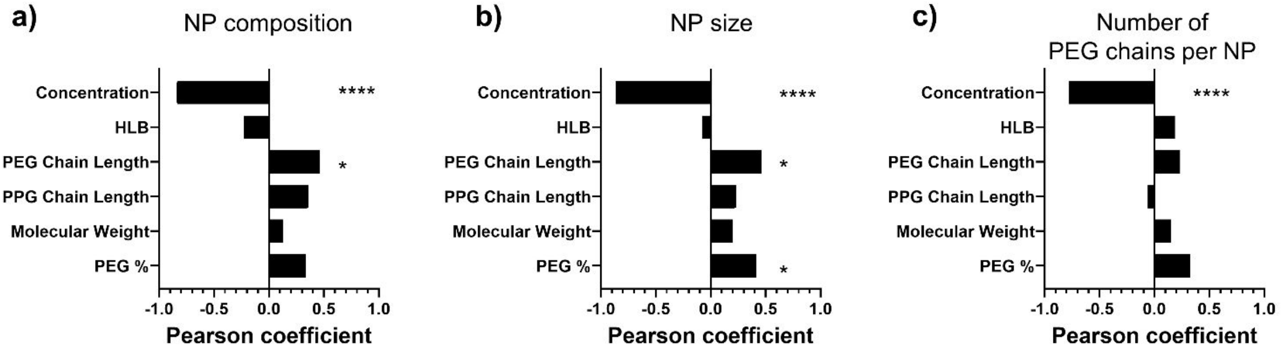

Figure S3. Correlation between copolymer properties on NP properties. a-c) Pearson coefficient for the NP properties a) composition (propylene sulfide units per NP/copolymer units per NP), b) size, and c) PEG chains per NP versus copolymer properties, with * representing significant correlation.

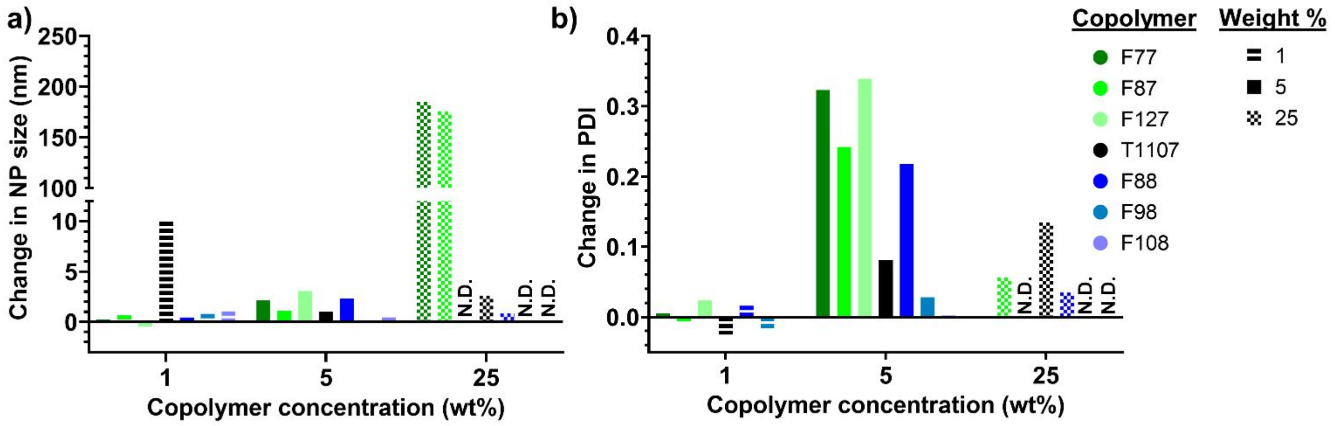

Figure S4. Nanoparticle stability. a) Change in nanoparticle hydrodynamic size after at least 40 days at 4°C. b) Change in nanoparticle polydispersity index (PDI) after at least 40 days at 4°C (n=1).

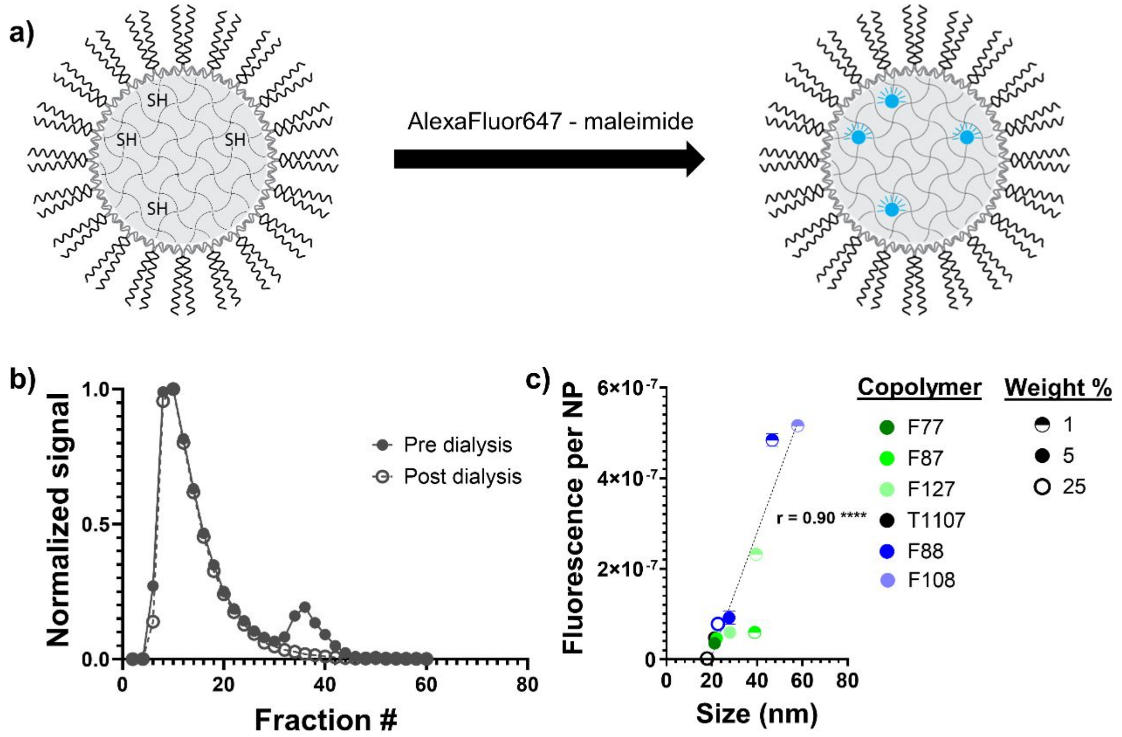

Figure S5. Fluorescently labeling the core of the NPs. a) Schematic of conjugating maleimide-functionalized AlexaFluor647 fluorophores to thiols in the NP core. b) Representative Sepharose CL-6B size-exclusion chromatography curve of AlexaFluor647 fluorescence before and after dialyzing the fluorophore-labeled NPs. c) Fluorescence intensity per NP compared to NP size. Dotted line represents linear regression with Pearson correlation coefficient (r), and **** indicates the slope significantly deviates from zero (n=1-2).

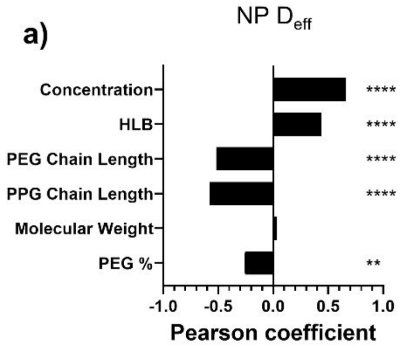

Figure S6. Correlation between copolymer properties and NP diffusivity. a) Pearson coefficient for the NP diffusivity versus copolymer properties, with * representing significant correlation.

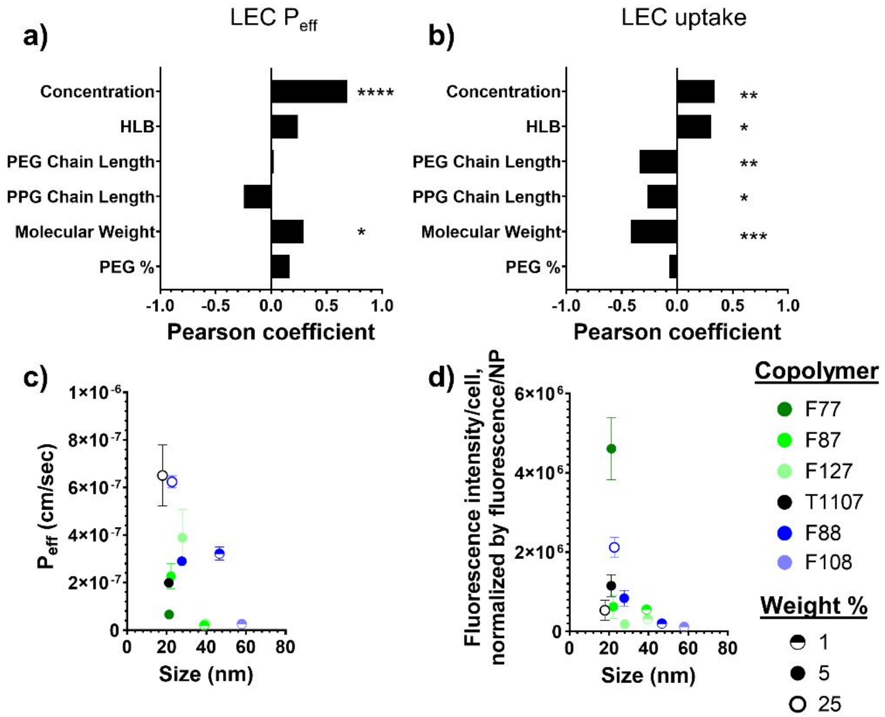

Figure S7. Correlation between copolymer properties and NP permeability. a-b) Pearson coefficient for the a) NP permeability and b) NP uptake versus copolymer properties, with * representing significant correlation. c-d) c) NP permeability (n=3-6) and d) uptake into LEC (n=4-6) dependence on NP size.
